# What Does Soluble C-Type Lectin-like Receptor 2 (sCLEC-2) × D-Dimer/Platelet (PLT) (sCLEC-2 × D-Dimer/PLT) Mean for Coagulation/Fibrinolysis Conditions? Comment on Yamamoto et al. Super Formula for Diagnosing Disseminated Intravascular Coagulation Using Soluble C-Type Lectin-like Receptor 2. *Diagnostics* 2023, *13*, 2299

**DOI:** 10.3390/diagnostics14010041

**Published:** 2023-12-25

**Authors:** Hiroyasu Ishikura

**Affiliations:** Department of Emergency and Critical Care Medicine, Faculty of Medicine, Fukuoka University, 7-45-1 Nanakuma, Jonan-ku, Fukuoka 814-0180, Japan; ishikurah@fukuoka-u.ac.jp; Tel.: +81-92-801-1011

I read with great interest the article by Akitaka Yamamoto et al. that was recently published in *Diagnostics* [1]. I congratulate the authors for their work, which contributes to the early diagnosis of disseminated intravascular coagulation (DIC) or pre-DIC using soluble C-type lectin-like receptor 2 (sCLEC-2), a novel platelet activation biomarker. An early diagnosis of DIC may enable earlier therapeutic intervention, resulting in an improved prognosis for patients.

My research group also previously reported the clinical utility of sCLEC-2 measurement for the diagnosis of sepsis-induced DIC (SID) [2]. I would like to offer the following remarks on the report by Yamamoto et al. [1] regarding the relationship between sCLEC-2 concentrations and patients with DIC.

Under the circumstance of pre-DIC or DIC, platelets are usually highly activated. However, because thrombocytopenia is frequently observed in DIC, the total plasma sCLEC-2 concentrations released from decreased platelets may not be sufficiently elevated in patients with DIC. Additionally, sCLEC-2 concentrations are likely to be affected by the platelet count. Therefore, this level could remain low under a low platelet count and would not increase immediately. Therefore, my research group proposed calculating the sCLEC-2/platelet count ratio (called the C2PAC index) to determine sCLEC2 concentrations per platelet unit and considered this index an index of platelet activation [2]. We concluded that the C2PAC index is a useful predictor of the progression and diagnosis of SID in patients with sepsis. Additionally, Ando et al. measured sCLEC-2 concentrations in patients undergoing neurosurgery for high-grade glioma and concluded that the C2PAC index is a potential marker that can detect postoperative venous thromboembolism formation [3].

Yamamoto et al. proposed the new “sCLEC-2 × D-dimer/PLT” [1]. They concluded that the sCLEC-2 × D-dimer/PLT formula is simple, easy, and highly useful for the diagnosis of DIC and pre-DIC without the use of a DIC scoring system. My research group previously performed a stepwise multiple logistic regression analysis to assess the relationship between SID and various molecular markers [2]. We reported that using the C2PAC index and D-dimer concentrations as predictive markers enabled the diagnosis of SID with a high probability (area under the curve (AUC), 0.95280; sensitivity, 0.9545; specificity, 0.8846) (Figure 1). We decided to calculate sCLEC-2 × D-dimer/PLT in the patient population enrolled in our previous study who were diagnosed with sepsis and were aged 18 years or older [2]. We used a receiver operating characteristic analysis to examine the diagnostic accuracy of DIC. We found a high AUC (0.9458), and the sensitivity and specificity were both 0.8846 (Figure 2). However, what sCLEC-2 × D-dimer/PLT represents regarding coagulation/fibrinolysis conditions is unclear. Although Yamamoto et al. [1] described how sCLEC-2 × D-dimer reflects the activation of platelets and coagulation, they did not explain what sCLEC-2 × D-dimer/PLT represents. I consider explaining what the value of sCLEC-2 × D-dimer divided by the platelet count reflects is important for the readership of *Diagnostics*.

Additionally, Yamamoto et al.’s study population included patients with post-cardiopulmonary arrest (n = 25) [1]. In all underlying diseases, except for patients with unidentified clinical syndromes, patients with post-cardiopulmonary arrest showed different platelet counts, values of coagulation/fibrinolytic markers, and DIC scores compared with those with other underlying diseases (Table 1) [1]. I feel this discrepancy might introduce research bias. Additionally, patients diagnosed with DIC should be analyzed based on underlying disease, such as solid cancer, hematological malignancy, aortic aneurysm, trauma, or infection. This perspective underscores the importance of meticulous consideration in research endeavors. I offer commentary on this study [1], which I believe could be of value to researchers grappling with the formulation of formulas within clinical studies.

## Figures and Tables

**Figure 1:**
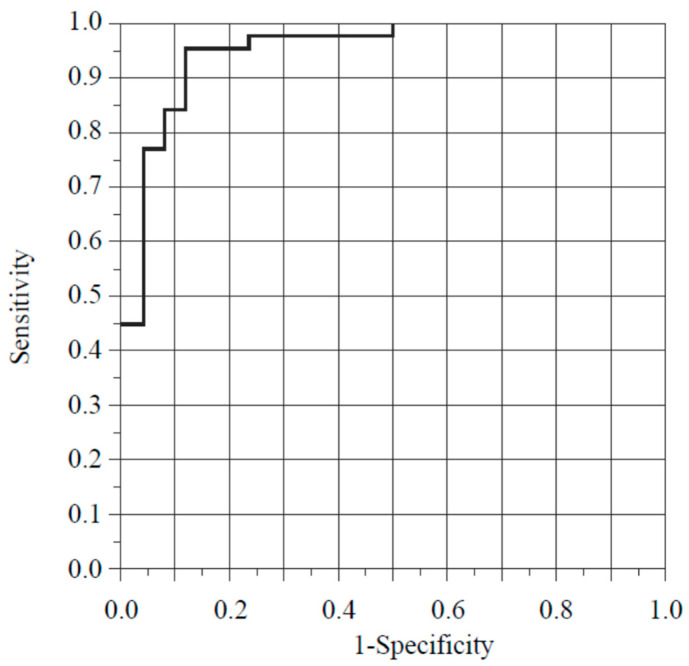
Receiver operating curve for predictive SID models in our case [2]. When the C2PAC index and D-dimer concentrations are selected as predictive markers for the diagnosis of SID, SID can be diagnosed with a high probability (AUC, 0.95280; sensitivity, 0.9545; specificity, 0.8846). C2PAC index means the ratio of sCLEC-2/platelet count. SID, sepsis-induced coagulopathy; sCLEC-2, soluble C-type lectin-like receptor 2; AUC, area under the curve.

**Figure 2:**
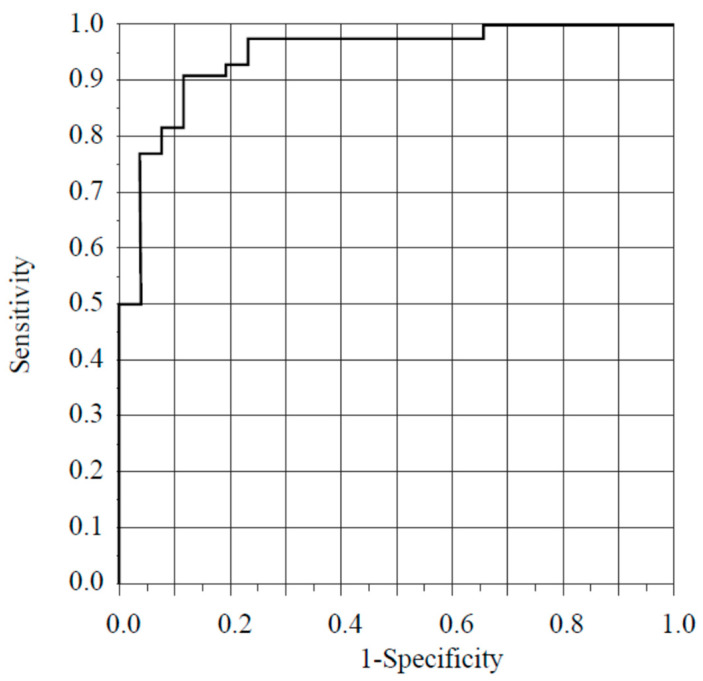
Receiver operating curve for predictive SID models in Yamamoto’s case [1]. When sCLEC-2 × D-dimer/PLT is selected as a predictive marker for the diagnosis of SID, SID can be diagnosed with a high probability (AUC, 0.9548; sensitivity and specificity, 0.8846). SID, sepsis-induced coagulopathy; sCLEC-2, soluble C-type lectin-like receptor 2; PLT, platelet count.

**Table 1:** Demographics and clinical characteristics, coagulation/fibrinolysis markers, DIC score and sCLEC2 in patients with each disease [1].

	Solid Cancer	HM	AA	Trauma	Infection	CPA	UCS
n	27	11	37	41	215	25	74
Age (years)	73.0 (69.0–79.8)	73.0 (58.0–83.0)	74.0 (67.0–78.0)	69.0 (42.0–80.3)	77.0 (61.0–83.0)	81.0 (68.8–87.3)	57.5 (48.0–73.0)
Sex (F:M)	12:15	4:7	17:20	20:21	99:116	8:17	39:35
Death (mortality)	8 (29.6%)	0 (0%)	3 (8.1%)	4 (9.8%)	25 (11.6%)	22 (88%)	0 (0%)
APTT (sec)	30.5 (27.0–35.0)	30.0 (27.0–35.8)	29.5 (28.0–34.5)	28.0 (26.0–34.0)	33.0 *** (29.0–39.0)	57.0 *** (44.3–76.0)	29.0 (27.0–32.0)
PT-INR	1.07 *** (1.02–1.21)	0.97 (0.94–1.26)	1.02 *** (0.97–1.18)	0.96 (0.92–1.05)	1.13 *** (1.03–1.24)	1.62 *** (1.21–1.97)	0.96 (0.92–1.00)
PLT (×10^9^/L)	226 (137–309)	191 (132–258)	160 *** (119–200)	227 (166–275)	191 *** (125–253)	114 *** (85–178)	23.4 (188–275)
DIC score	2.0 *** (1.0–4.8)	1.0 *** (1.0–3.8)	3.0 *** (1.0–4.0)	1.0 *** (1.0–4.0)	2.0 *** (1.0–4.0)	7.0 *** (5.0–8.0)	0 (0–0)
FDP (μg/mL)	4.4 *** (2.8–28.3)	4.6 ** (1.2–17.9)	3.7 *** (0.6–7.5)	8.5 *** (3.3–37.8)	5.6 *** (2.4–14.5)	67.6 *** (22.2–452.8)	0.7 (0.3–1.0)
D-dimer (μg/mL)	3.8 *** (1.9–16.6)	4.1 ** (0.7–11.9)	6.8 *** (2.6–11.6)	7.6 *** (2.4–18.0)	4.4 *** (1.6–9.7)	15.7 *** (7.6–46.4)	0.6 (0.4–1.5)
SF (μg/mL)	6.7 *** (2.2–13.4)	5.8 (3.0–21.1)	8.1 *** (1.6–12.7)	6.8 *** (1.1–9.1)	9.6 *** (2.0–15.0)	12.7 *** (2.1–25.5)	2.1 (0.5–4.7)
sCLEC2 (ng/L)	260 ** (172–321)	278 * (167–364)	247 *** (174–323)	245 *** (178–328)	258 ** (195–335)	441 *** (310–748)	193 (143–242)

Data are shown as the median (25th–75th percentiles). HM, hematological malignancy; AA, aortic aneurysm; CPA, cardiopulmonary arrest; UCS, unidentified clinical syndrome; F:M, Female:Male; APTT, activated partial thromboplastin time; PT-INR, prothrombin time-international normalized ratio; PLT, platelet count; DIC, disseminated intravascular coagulation; FDP, fibrinogen and fibrin degradation products; SF, soluble fibrin; sCLEC-2, soluble C-type lectin-like receptor 2. * *p* < 0.05; ** *p* < 0.01; and *** *p* < 0.001 compared with UCS. The subject study protocol (2019-K9) was approved by the Human Ethics Review Committee of Mie Prefectural General Medical Center, and informed consent was obtained from each participant. This study was carried out in accordance with the principles of the Declaration of Helsinki.

## Data Availability

Data are available at https://doi.org/10.1080/09537104.2021.2019694, accessed on 10 September 2023.
